# Draft Genome Sequence of *Bacillus subtilis* strain KATMIRA1933

**DOI:** 10.1128/genomeA.00619-14

**Published:** 2014-06-19

**Authors:** Andrey V. Karlyshev, Vyacheslav G. Melnikov, Michael L. Chikindas

**Affiliations:** a School of Life Sciences, Faculty of Science, Engineering and Computing, Kingston University, Kingston upon Thames, United Kingdom; b International Science and Technology Center, Moscow, Russia; c School of Environmental and Biological Sciences, Rutgers State University, New Brunswick, New Jersey, USA; d Astrabiol LLC, Highland Park, New Jersey, USA

## Abstract

In this report, we present a draft sequence of *Bacillus subtilis* KATMIRA1933. Previous studies demonstrated probiotic properties of this strain partially attributed to production of an antibacterial compound, subtilosin. Comparative analysis of this strain’s genome with that of a commercial probiotic strain, *B. subtilis* Natto, is presented.

## GENOME ANNOUNCEMENT

*Bacillus* sp. strain KATMIRA1933 was isolated from the fermented dairy product YoguFarm (catalog no. 74699-02905; JSL Foods, Los Angeles, CA, USA) (1). The microorganism was consistently isolated from this dairy product from 2007 to 2009, despite its absence from the list of the product’s ingredients. Due to the lack of any records of adverse health issues associated with consumption of the product, the strain is likely to be safe. Based on 16S ribosomal RNA analysis performed (Accugenix, Newark, DE, USA), the microorganism was initially identified as *B. amyloliquifaciens* (1). However, based on comparative genomics analysis, we identified this strain as *B. subtilis*. This strain’s subtilosin was found to possess spermicidal activity (2). It was active against food-borne (3) and vaginal (4) pathogens, and against HSV-1 (5).

The sequencing data were generated using an IonTorrent PGM and 314v2 chip. While 88.22% of reads could be mapped onto the genome of *B. subtilis* reference strain BSP1, only 36.39% of them could be mapped onto a reference strain of *B. amyloliquefaciens* LFB112.

Using IonTorrent Assembler, the reads were assembled into 125 contigs (1 kb to 214 kb) with 22.45× genome coverage. The total size of the assembly (4,263,792 bases) and G+C content (43.4%) were in good agreement with respective figures for other strains of *B. subtilis* (4.01 to 4.22 Mb and 43.5 to 43.9%).

The genome of test strain was compared with that of *B. subtilis* Natto Best 195 (6) using read mapping and CLC Genomics Workbench software. Present in the latter but not in the former were the genes encoding a chitinase, particular transposases and a spore coat protein X. Genes *comX* (encoding a quorum-sensing pheromone precursor), *comQ* (encoding a quorum-sensing protein), *degQ* (regulating hyperproduction of levansucrase and other extracellular degradative enzymes), and some bacteriocin transport-related genes were missing in KATMIRA1933, although bacitracin transport-related genes were detected.

Genome annotation using the RAST server (7) identified 4,417 protein-encoding sequences. Structural genes coding for resistance to vancomycin, fluoroquinolones, fosfomycin, and beta-lactam antibiotics, as well as mulidrug resistance efflux pumps-encoding genes were detected. A gene encoding a secreted Zn peptidase with similarity to aureolysin and bacillolysin was also present in the genome of KATMIRA1933. The strain also contains nattokinase (subtilisin) and subtilosin encoding genes (99% and 100% amino acid sequence identity to Natto strain’s products, respectively).

Among the genes found in KATMIRA1933 but absent in strain Natto were those involved in polyglycerol phosphate biosynthesis, a gene encoding tyrocidine/plipastatin synthetase (with low similarity to gramicidin and fengycin synthetases of *B. amyloliquefaciens* and fusaricidin synthetase of *Paenibacillus* spp.), and a gene encoding a putative extracellular protease with 100% identity to the protein produced by *B. subtilis* BSn5 and 53% identity to bacillolysin of *B. cereus* spp.

The availability of sequence of *B. subtilis* KATMIRA1933 will assist in better understanding the mechanisms of its probiotic action and in the development of novel strains with improved properties.

### Nucleotide sequence accession numbers.

This whole-genome shotgun project has been deposited at DDBJ/EMBL/GenBank under the accession no. JMEF00000000. The version described in this paper is version JMEF01000000.
